# Thyroid Hormones and Antioxidant Systems: Focus on Oxidative Stress in Cardiovascular and Pulmonary Diseases

**DOI:** 10.3390/ijms141223893

**Published:** 2013-12-09

**Authors:** Antonio Mancini, Sebastiano Raimondo, Chantal Di Segni, Mariasara Persano, Giovanni Gadotti, Andrea Silvestrini, Roberto Festa, Luca Tiano, Alfredo Pontecorvi, Elisabetta Meucci

**Affiliations:** 1 Department of Medical Sciences, Division of Endocrinology, Catholic University; Rome 00168, Italy; E-Mails: bastio984@hotmail.com (S.R.); chantal_ds86@hotmail.com (C.D.S.); sarapers@hotmail.it (M.P.); giovanni.gadotti@gmail.com (G.G.); pontecorvi@rm.unicatt.it (A.P.); 2 Institute of Biochemistry and Clinical Biochemistry, Catholic University, Rome 00168, Italy; E-Mails: asilvestrini@rm.unicatt.it (A.S.); emeucci@rm.unicatt.it (E.M.); 3 Department of Clinical and Molecular Science, Polytechnic University of the Marche, Ancona 60100, Italy; E-Mail: festa7r@libero.it; 4 Department of Clinical and Dental Sciences, Polytechnic University of Marche, Ancona 60100, Italy; E-Mail: l.tiano@univpm.it

**Keywords:** oxidative stress, coenzyme Q_10_, total antioxidant capacity, thyroid hormones, “low-T3” syndrome

## Abstract

In previous works we demonstrated an inverse correlation between plasma Coenzyme Q_10_ (CoQ_10_) and thyroid hormones; in fact, CoQ_10_ levels in hyperthyroid patients were found among the lowest detected in human diseases. On the contrary, CoQ_10_ is elevated in hypothyroid subjects, also in subclinical conditions, suggesting the usefulness of this index in assessing metabolic status in thyroid disorders. A Low-T3 syndrome is a condition observed in several chronic diseases: it is considered an adaptation mechanism, where there is a reduction in pro-hormone T4 conversion. Low T3-Syndrome is not usually considered to be corrected with replacement therapy. We review the role of thyroid hormones in regulation of antioxidant systems, also presenting data on total antioxidant capacity and Coenzyme Q_10_. Published studies suggest that oxidative stress could be involved in the clinical course of different heart diseases; our data could support the rationale of replacement therapy in low-T3 conditions.

## 1. Introduction

It is well known that oxidative stress (OS), defined as an imbalance between radicals and antioxidant defense, is implicated as a pathophysiological mechanism of different diseases and is a topic of growing interest [1]. Cell injury is a consequence of OS; recognized targets are DNA, lipids and proteins, which react with hydroxyl radicals to form specific products [2]. Especially in the field of cardiovascular diseases, the role of OS has been revaluated [3], even if the therapeutic aftermath is still debated. Antioxidant defenses include enzymatic and non-enzymatic molecules and they are modulated by hormones, which regulate their synthesis and turnover as previously reviewed [4]. In previous papers, we focused our attention on Coenzyme Q_10_ (CoQ_10_), a lipophilic antioxidant, with a key role in energy metabolism, showing its alteration in thyroid and pituitary disorders [5,6]. It is also called ubiquinone because of its ubiquitous diffusion in organisms and tissues. It is a key component of the mitochondrial oxidative phosphorylation chain as a link between flavoproteins and cytochromes in the inner mitochondrial membrane. It also has many other functions, first of all a powerful antioxidant activity, and new roles in different cellular functions are continuously discovered. This molecule can participate in oxido-reductive reactions in mitochondria, in lysosomes, in the Golgi apparatus and plasma membranes [7]; it also contributes to membrane fluidity. Moreover CoQ_10_ can take part in many aspects of the oxido-reductive control of cellular signalling origin and transmission; in fact the auto-oxidation of semi-quinon, formed in various membranes during electron transport, can be a primary source for the H_2_O_2_ generation, which activates transcription factors, e.g., NF-κB, to induce gene expression [8]. It is also possible that ROS generation could suppress other genes reinforcing the role of antioxidants in gene regulation.

Both hypothyroidism and hyperthyroidism can be associated with OS, moreover thyroid hormone (TH)-induced oxidative damage could be a factor responsible for the progression of heart failure, as suggested by the benefit of T_3_ administration on antioxidant systems in rat heart after pharmacological-induced hypothyroidism [9]. However, few data exist on the possible diagnostic role of antioxidant measurements; in this review we examine thyroid regulation of antioxidants and OS in cardiac physiology and disease; then we speculate on the situation of low-T_3_ syndrome (also called “non-thyroidal illness”, NTIS) [10], a condition present in chronic disease. This hormonal situation reflects a compensatory mechanism, but the need of replacement therapy is matter of discussion. Therefore the evaluation of OS parameters could represent a further insight into the pathophysiology of NTIS.

## 2. Thyroid Hormones and Oxidative Stress

Previous studies suggested that the hypermetabolic state of hyperthyroidism is associated with an increase in free radical production [11,12], while the hypometabolic state of hypothyroidism symmetrically leads to a reduced free radical production [13]. Indeed both hyperthyroidism and hypothyroidism are associated with enhanced oxidative stress involving enzymatic and non-enzymatic antioxidants [14]. Furthermore, some complications of hyperthyroidism are specifically related to the oxidative stress in target tissues [15]. Thyroid hormones can *per se* act as oxidants and produce DNA-damage (contrasted by catalase), probably through the phenolic group, similar to that of steroidal estrogens [16]. Many other mechanisms, reviewed by Venditti and Di Meo [17], can be involved: enhanced nitric oxide (NO)-Synthase (NOS) gene expression with NO overproduction; activation of hepatic NF-κB and the consequent increase of cytokines stimulating ROS generation; uncoupling mechanisms involving UCP-2 and UCP-3, regulated by thyroid hormones; increased turnover of mitochondrial proteins; mitoptosis, regulated by peroxisome proliferator-activated receptor gamma coactivator-1, which is upregulated by T_3_ administration. Thyroid hormones influence lipid composition of rat tissues [18] and therefore the susceptibility to oxidative stress.

However, there is specificity in tissue response, and differential effects of T_3_ and T_4_ are possible, as previously reviewed [19]. In rat liver, T_3_-induced hyperthyroidism was found to be associated with altered lipid-peroxidation indices, including elevated levels of thiobarbituric acid reactive substances (TBARS) and hydroperoxides [11,20–22]. On the contrary, no change in TBARS was observed in homogenized livers from rats made hyperthyroid by administration of T4 over a 4-week period [13]. As regards testis, no significant change was observed in lipid peroxidation (evaluated as TBARS or hydroperoxides) of hyperthyroid adult rats, but hyperthyroidism promoted protein oxidation rate as indicated by an enhanced content of protein-bound carbonyls [23]. In conclusion, it is important to emphasize the fact of a tissue-linked variability in the effects of hyperthyroidism on the activity of antioxidant enzymes (Mn-superoxide dismutase (SOD) or Cu,Zn-SOD, catalase (CAT), glutathione-peroxidase) with differential effects of the two thyroid hormones [17].

The model of cardiac hypertrophy induced by experimental hyperthryroidism in rats has been recently investigated: a T4 treatment in male Wistar rats induced an increase in the left ventricular end-diastolic pressure, coupled with increase of protein oxidation, H_2_O_2_, NO metabolites and decrease of GSH/GSSG ratio, vitamin C, total radical trapping antioxidant potential, suggesting the role of oxidative stress in such a model. Vitamin E attenuated these alterations. The involvement of redox activation of AKT1 and JUN/FOS signaling was also demonstrated [24]. The same group also showed that the thyroid-induced cardiac hypertrophy is mediated by angiotensin receptors I and II activation [25]. Also in this case antioxidant administration ameliorated gene and protein expression of angiotensin II receptors and cardiac hypertrophy. Also hyperthyroidism-related hypertension is related to oxidative stress. In male Wistar rats, the administration of tempol, which is a cell membrane-permeable SOD mimetic, reduced blood pressure acting via antioxidant mechanisms, as demonstrated by a decrease of plasma malondialdehyde (MDA) and urinary excretion of F2 isoprostanes in hypertensive animals, but not in controls [26]. Tempol also increased the slopes of the relationships between renal perfusion pressure and natriuresis [27].

In humans, hyperthyroidism has been associated with reduced circulating levels of alpha-tocopherol [28,29] and Coenzyme Q_10_[29,30]. Coenzyme Q_10_ showed an increasing trend in hypothyroidism [30] and it appeared to be a sensitive index of thyroid hormones effect, in situations like drug interference [31], or systemic illness where a low-T3 condition [32] could complicate the interpretation of thyroid hormone levels (see discussion below). Few data are available on CoQ_10_ levels in human tissues; they seem to be similar to those in plasma: in active proliferating tissue (toxic goiter or neoplasias) CoQ_10_ concentrations were greater than in unaffected areas [30]. This phenomenon points to an increased CoQ_10_ synthesis related to the increased metabolic requests; it is in agreement with the increased levels of CoQ_9_ in liver mitochondria from rats rendered mildly hyperthyroid [33].

However, data on hypothyroidism in humans are conflicting. Baskol *et al.* showed in a group of 33 patients with primary hypothyroidism elevated MDA and NO levels and low paraoxonase (PON1) activity, while SOD was not different from controls. Interestingly, thyroid treatment decreased MDA and increased PON1, without reaching levels observed in controls [34]. They concluded that a pro-oxidant environment in hypothyroidism could play a role in the pathogenesis of atherosclerosis in these patients. Elevated MDA levels were also observed in subclinical hypothyroidism [35]; an increased OS was attributed to lack of antioxidants but also to altered lipid metabolism, since MDA showed a correlation with LDL-cholesterol, total cholesterol and triglycerides. Total antioxidant status was similar in overt hypothyroidism, subclinical hypothyroidism and controls.

Different studies confirmed the NO elevation in hypothyroid patients [36,37]. Data on other parameters are conflicting. As PON-1 is concerned, a decreased activity was observed both in hypo- and hyperthyroidism [38], while significant differences were not seen with controls in other studies [36].

Another study [39] showed increased levels of TBARS, but also of antioxidants, such as SOD, CAT and vitamin E. All these parameters correlated with T_3_; moreover the correlation between T_3_ and CAT remained significant also when corrected with total cholesterol. While TBARS elevation was also shown in some studies [37,40], other studies did not confirm the data in overt hypothyroidism [36] and in subclinical hypothyroidism [41].

We showed low Total Antioxidant Capacity (TAC) levels in hypothyroid patients [5] and increased CoQ_10_ levels also in secondary hypothyroidism (mainly due to its metabolic role in mitochondrial respiratory chain and therefore underutilized in hypothyroid tissue). In the last case, hypothyroidism has a predominant effect on other conditions that lead to a decrease in CoQ_10_ levels, such as acromegaly, hypo-adrenalism and hypogonadism [5,42,43].

Finally, new perspectives concern DUOX (Dual Oxidase) gene expression, which is crucial for H_2_O_2_ generation essential for thyroid peroxidase (TPO)-catalyzed thyroid hormone synthesis [44]. Two oxidases of this family are present in thyroid (DUOX1 and DUOX2) and work together with maturation factors (DUOXA1 and DUOXA2), which allow DUOX proteins to translocate to the follicular cell membrane and exert their enzymatic activity. Cases of hypothyroidism due to mutation of DUOX or DUOXA genes have been presented in the literature [45,46]. While defects of this system interfere with thyroid hormone synthesis, another new intracellular ROS generating system has been demonstrated in the human thyroid gland: NADPH oxidase 4 (NOX4) [47]; defects in such a system could be associated with thyroid cancer (via activation by the H-Ras oncongene) and Hashimoto’s thyroiditis (in such a situation an increased extracellular ROS production causes an augmented ICAM-1 expression and cytokine release) [48].

Since hyperlipidemia too can induce oxidative stress, as demonstrated in animals and humans [49,50], thyroid hormone effects could be also mediated by interference with lipid metabolism. Lipoprotein plasma levels increase in hypothyroidism, together with a reduction of oxidative metabolism [51,52]. Hypothyroid patients present higher lipoperoxide (LPx) levels, a significant higher LDL content in the lipid peroxides and higher oxidation rate; they also exhibit elevation in β-carotene levels with higher LDL oxidation [53]. Finally oleic to linoleic acid ratio, which is inversely proportional to oxidative stress, is lower in hypothyroidism [54].

## 3. Thyroid Hormones, Antioxidants and the Heart

ROS have been indicated as both detrimental and protective, via different pathways, for cardiac myocyte functions, electrophysiology and pharmacology. ROS effects on contractility is well recognized in literature, but recently also cardiac excitability has been investigated [55]. ROS influence sarcolemmal and mitochondrial ion channels, which are responsible for cardiomyocyte excitability.

It is known from the literature that oxidative stress is involved in the clinical course of different cardiopathies and in general it is involved in negative outcomes in cardiovascular disease [56,57]. ROS have a crucial role in the genesis of atherosclerosis inducing vascular smooth muscle cell (SMC) growth and proliferation, oxidation of LDL, reduction of NO bioavailability, and vascular inflammation, which are characteristic features of the disease [9]. Oxidative stress is also important in myocardial remodeling after a myocardial infarction, inducing fibroblast proliferation and collagen synthesis [9]. Patients with dilatative cardiomyopathies have increased oxidative stress, in particular their erythrocyte membranes show an augmented sensitivity to the lipoperoxides and oxidative damage [9]. The worsening of ventricular dysfunction recognizes as a possible factor myocyte apoptosis related to increased ROS formation [58].

On the other hand, CoQ_10_ administration has been shown to be useful in the treatment of cardiomyopathies. Therefore a link could be present between TH, antioxidant and cardiac function [59].

In previous works we demonstrated an inverse correlation between plasma Coenzyme Q_10_ and thyroid hormones, with CoQ_10_ levels in hyperthyroid patients among the lowest discovered in human diseases [30]. On the contrary, CoQ_10_ is elevated in hypothyroid subjects, also in subclinical conditions, suggesting the usefulness of this index in assessing metabolic status in thyroid disorders [31]. This correlation is so common that it makes CoQ_10_ determination a useful index in clinical situations in which hormone values do not correlate with the metabolic status of the patients. Indeed in Amiodarone treated subjects the drug invariably alters the indexes usually employed to measure thyroid function; in this situation CoQ_10_ correlates with the metabolic state better than with thyroid hormone levels themselves [31]. The possible explanations for the very low CoQ_10_ levels in hyperthyroid patients include: decreased synthesis related to competition for tyrosine, which is a common substrate for CoQ or thyroxine synthesis, even if this hypothesis is disconfirmed by experimental data in animals; increased CoQ_10_ utilization, due to the increased stimulation of energy metabolism; increased degradation; decreased levels of carriers in serum, since it has been demonstrated that the release of VLDL from liver is decreased in hyperthyroid states; similar mechanisms can be invoked to explain high CoQ_10_ levels in hypothyroid patients [30]. An important index of body antioxidant defense is the antioxidant capacity of blood plasma, which is studied more and more frequently. Representing the functional sum of antioxidants present in plasma, it is a measure of the extracellular antioxidant barrier [60–62]. In a recent work, TAC was determined during cardiovascular bypass surgery in patients with coronary heart disease: TAC decreased during surgery, but no further decrease in TAC was observed during reperfusion, indicating that it is a relatively stable parameter of the antioxidative barrier of the body [63].

We have determined total antioxidant capacity to appraise if variations in CoQ_10_ levels are parallel to those of the other antioxidant systems in different subsets of thyroid disorders. We have studied 73 subjects of whom 25 were hyperthyroid patients, 27 hypothyroid patients and 21 normal subjects.

A blood sample, obtained at 08:00 a.m., after overnight fasting, was collected in order to measure thyroid hormones, lipid values and CoQ_10_ concentration. The hormones were determined by RIA method. Normal values of the studied hormones are: fT_3_: 2.3–4.2 pg/mL; fT_4_: 8.5–15.5 pg/mL; TSH: 0.35–2.80 μUI/mL. CoQ_10_ levels were measured with a standardized method by the International CoQ_10_ Association, using HPLC as previously described [64]. The normal value of CoQ_10_ is 0.7–1 μg/mL. Moreover, the CoQ_10_ results were related to plasma cholesterol concentration.

Total Antioxidant Capacity (TAC) was evaluated as previously described [65], with a modification of the method developed by Rice-Evans and Miller [66]. The method is based on the antioxidants inhibition of the absorbance of the radical cation 2,2^I^-azino-bis (3-ethylbenzothiazoline-6 sulphonate) (ABTS^•+^) formed by interaction between ABTS (150 μM) and ferrylmyoglobin radical species, generated by activation of metamyoglobin (2.5 μM) with H_2_O_2_ (75 μM). The presence of chain-breaking antioxidants induces a lag time (the Lag phase) in the accumulation of ABTS^•+^ whose duration is proportional to the concentration of this type of antioxidant. Antioxidant capacity afforded by chain-breaking antioxidants is expressed as length of Lag phase (LAG, sec). In the LAG mode, the assay mainly measures non-protein and non-enzymatic antioxidants that are primarily extracellular chain-breaking antioxidants, such as ascorbate, urate and glutathione [65].

**Figure 1.**
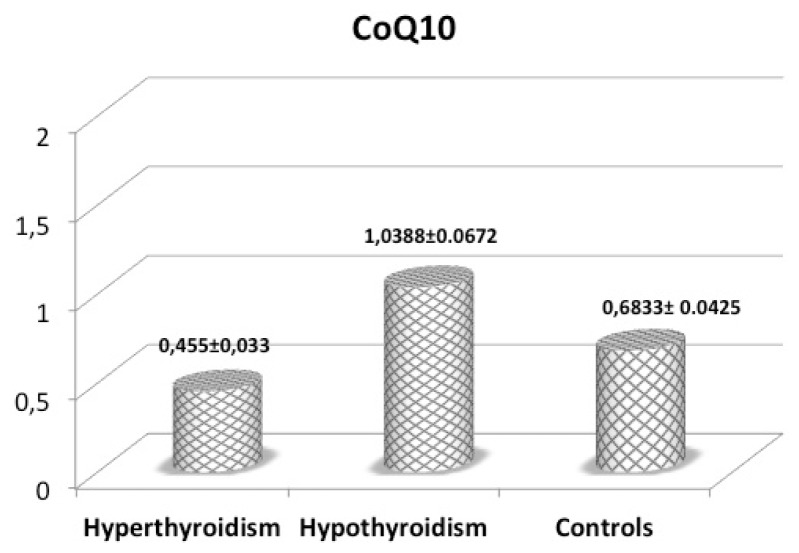
Mean (±SEM) plasma CoQ_10_ levels (μU/mL) in patients with hyperthyroidism, hypothyroidism and control subjects.

**Figure 2.**
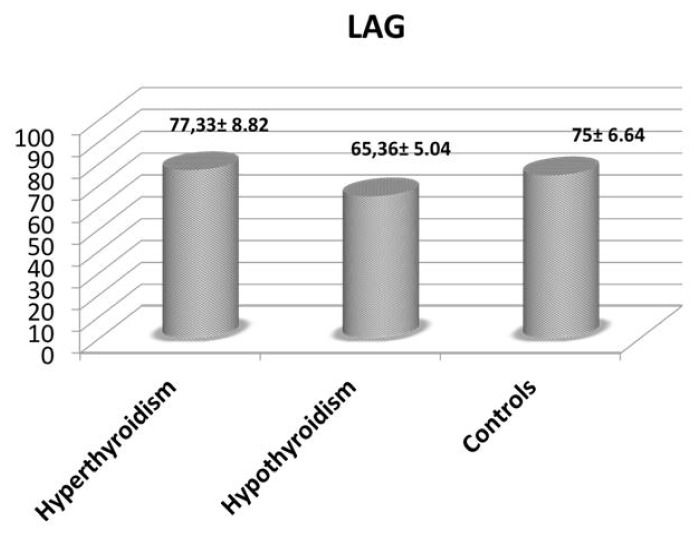
Mean (±SEM) LAG values (sec) in patients with hyperthyroidism, hypothyroidism and control subjects.

We found a correlation between the values of fT_4_ and the CoQ_10_ (*p* = 0.00) as well as between fT_3_ and the CoQ_10_ (*p* = 0.00) We have not found meaningful correlations between fT_3_ and CoQ_10_ corrected for the values of cholesterol and between fT_4_ and CoQ_10_ corrected for the values of cholesterol (*p* = 0.336 and *p* = 0.396 respectively). Mean CoQ_10_ and LAG values are reported in Figures 1 and 2; we observed a different pattern in these two parameters. While LAG values showed a trend toward lower levels in hypothyroidism, suggesting reduced antioxidant defenses, CoQ_10_ exhibited the highest levels (significantly different from those in hyperthyroidism) in hypothyroid patients, confirming that CoQ_10_ levels better express the sensitivity of tissues to TH, and therefore a lower tissue utilization in hypothyroidism. This could have essential consequences in the cases of NTIS, and especially on heart and lung function.

## 4. Low-T3 Syndrome

In critical illness, several abnormalities in TH secretion, metabolism and action have been described in patients without previous diagnosis of intrinsic thyroid disease and are collectively called “Non thyroidal illness syndrome” (NTIS) [10,67]; this term is now largely employed, instead of “euthyroid sick syndrome” [68,69] or “low-T_3_ syndrome”. The last term refers to the most common abnormality, a decreased level of serum total triiodothyronine (T_3_), which can be detected very early, within 2 h after the onset of severe physical stress [70–73]. However, T_3_ lowering is only one characteristic of the endocrine picture described is this situation; therefore the term NTIS seems to be more appropriate, also strengthening its extra-thyroidal source.

NTIS has been depicted in about 70% of hospitalized patients for different diseases [74–76]. Moreover, the severity of morbidity and outcome in patients studied in the intensive care unit (ICU) has been correlated with the alteration in thyroid function [77,78]. The hormonal response exhibits different patterns in acute and chronic phase, since in the first phase alterations prevail in peripheral metabolism of TH, while in the following phase central mechanisms controlling thyroid secretion progressively arise [79,80].

Since there is no clear evidence of tissue hypothyroidism, this condition seems to be an adaptive response, and thyroid replacement therapy is not usually required, but this topic is still debated, since indirect signs of true hypothyroidism at tissue levels have been shown [81]. The question is open and different reviews have been published on this topic [67,82–86]; experimental data suggest a condition of hypothyroidism in NTIS, such as the model of salt loading-induced hypertension in rats, which is prevented by clofibrate, due to its anti-thyroid action [87]. Recently different molecular mechanisms have been investigated to define the complex pathogenesis of NTIS. The role of intracellular oxidative stress has been underlined [67,88].

As previously reviewed [67], a low T_3_ state has been described in a variety of clinical situations, such as starvation [89], sepsis [90], surgery [91], trauma [92], myocardial infarction and heart failure [93,94], cardiopulmonary bypass [95], respiratory failure [96], bone marrow transplantation [97], and other severe illness [98]. In a recent paper in unselected ICU patients, free T_3_ (fT_3_) was the most powerful and the only independent predictor of ICU mortality, with a prognostic improving value when added to the APACHE II score [99]. A retrospective study in a large group of patients treated with mechanical ventilation (MV) confirmed that NTIS represents a risk factor for prolonged MV [100].

Due to the importance of TH in cardiac function, it is not surprising that cardiac patients have been extensively studied under this profile. TH influence cardiac function with different mechanisms: inotropic and chronotropic positive effect via nuclear and non-nuclear pathways in cardiomyocytes, increase in cardiac contractility through augmented tissue oxygen delivery and consumption; decrease in systemic vascular resistance, through direct TH action on vascular smooth muscle cells; other endocrine effects are exerted on renin-angiotensin-aldosterone axis and on erythropoietin secretion [85,101]. There is no evidence of conversion of T4 to T3 in cardiomyocytes [102]; however, thyroid hormones regulates expression of specific cardiomyocytes genes: some T3 responsive genes include the myosin heavy chains, phospholamban and sarcoplasmic reticulum calcium-activated ATPase, which are important for cardiac contractility [9,103].

One of the early studies was performed in patients serially followed after acute myocardial infarction; a sustained and prolonged decrease of total T_3_ and fT_3_ was described, while total T_4_ but not fT_4_ showed a transient decrease; thyroxine binding globulin (TBG) levels remained unchanged, while thyroxine-binding pre-albumin (TBPA) and albumin exhibited a prolonged fall. TSH, despite low T_3_, did not increase, remaining inappropriately low [104]. In this sense, the increase of TSH was shown to be correlated with a good prognosis [105].

It has been reported that patients with heart failure have low T_3_ serum concentrations, which correlates with cardiac function [106]. In advanced heart failure, a low fT_3_ index/reverse T_3_ ratio was associated with a higher right atrial pulmonary artery and pulmonary capillary wedge and lower ejection fraction [94].

Low T_3_ syndrome has been considered a strong predictor of death and directly implicated in poor prognosis of cardiac patients in a large group of patients admitted in a cardiology department [107].

Data in pulmonary, kidney and liver diseases are elsewhere reviewed. Different pathophysiological mechanisms are involved including: central suppression of TSH, altered TH blood transportation; expression and/or activity of deiodinases; thyroid receptors and post-receptorial mechanisms. A special role has been attributed to cytokines [10,67].

Interventional studies, reviewed by Bello *et al.* [85], show overall beneficial effects on cardiovascular parameters, but not unequivocal benefit in patients’ outcome. In fact, in patients with dilated cardiomyopathy, the administration of TH significantly increased left ventricular end-diastolic volume and stroke volume while it decreased heart rate [108]. In patients studied after coronary artery bypass surgery, the administration of intravenous T_3_ or placebo produced an increase in cardiac output and lowered systemic vascular resistance, without influencing the patients’ outcome and therapeutic schedules [109]. In contrast, another study [110] performed after elective coronary artery bypass grafting showed a beneficial effect of intravenous T_3_ administration on incidence of postoperative myocardial ischemia and on need for pacemakers or mechanical cardiac support devices.

Preliminary data of our group showed low T3 levels concurrently with signs of tissue hypothyroidism (elevated CoQ_10_ levels) in patients studied after major heart surgery [111]. In fact we found CoQ_10_ levels in the hypothyroid range, despite the fact that cardiac diseases are well known to be associated with low CoQ_10_. Since data suggest a tissue hypothyroidism, in NTIS, we think that OS could be enhanced by low T3 levels.

Some analogies do exist with another situation of NTIS, the chronic obstructive pulmonary disease (COPD). We have recently studied patients with COPD [81], evaluating lung parameters and antioxidant parameters, because of a possible involvement of OS in NTIS, as discussed above. COPD is a complex condition, which cannot be considered a lung-related disorder, but rather a systemic disease also associated to increased oxidative stress. We evaluated thyroid hormones and antioxidant systems, the lipophilic CoQ_10_ and total antioxidant capacity (TAC) in COPD patients to reveal the presence of a low-T3 syndrome in COPD and investigate the correlation between thyroid hormones, lung function parameters and antioxidants. The evaluation of CoQ_10_ was particularly interesting, also for the energetic role of this molecule, which is a component of the mitochondrial respiratory chain, as above stated; its concentrations were also corrected for cholesterol, due to its lipophilic nature. We studied 32 COPD patients and 45 controls; CoQ_10_ was assayed by HPLC; TAC by the metmyoglobin-ABTS method and expressed as latency time (LAG) in radical species appearance. We found significantly lower LAG values, fT3 and fT4 levels and significantly higher TSH in COPD patients *vs*. controls. LAG values significantly correlated with fT3 concentration. Twelve out of 32 patients exhibited fT3 levels lower than normal range. Dividing COPD patients in two groups on the basis of the fT3 concentration (normal fT3 COPD and low fT3 COPD), we observed lower LAG values in normal fT3-COPD, compared to healthy subjects, with a further significant reduction in low fT3-COPD patients. Moreover higher TSH concentrations were present in normal fT3-COPD, compared to healthy subjects, with a further significant increase in low fT3-COPD patients. CoQ_10_/cholesterol ratio was higher in low fT3-COPD *vs*. normal fT3-COPD, with a nearly significant difference. These data seem to indicate an increased oxidative stress in low fT3-COPD and a role of fT3 in modulating antioxidant systems. However low fT3 levels are coupled with metabolic indexes of true hypothyroidism, suggesting that elevated CoQ_10_ expresses a reduced tissue utilization. Interestingly, there was no significant difference in lung parameters when comparing normal- or low-fT3 COPD patients, according to the definition of COPD as a systemic disease, with respiratory parameters unable to define the severity of disease. In fact metabolic dysfunctions (*i.e.*, osteoporosis, vascular and cardiac involvement, muscle impairment) play a role in the natural history of disease, but they were found poorly related to respiratory impairment, underlying the need of indexes related to a real tissue condition; the pattern of fT3 could indicate such a situation, as reinforced by the pattern of CoQ_10_ levels; decreased plasma antioxidant capacity and increased CoQ_10_ levels in low fT3-COPD, again suggested a possible condition of hypothyroidism at tissue levels.

COPD represents another situation with alteration in thyroid hormone concentrations, not clearly related to metabolic status; similarly to what is found in other chronic diseases (kidney or liver failure, chronic inflammation, *etc.*) low T3 syndrome is considered an adaptation mechanism rather than a true hypothyroidism. Our preliminary data seem to indicate that low T3 levels are accompanied by metabolic indexes of a true hypothyroidism in COPD patients since CoQ_10_ levels are higher in this group of patients. Further studies are required in order to ascertain whether this data supports the need for hormone placement therapy in such a condition.

## 5. Conclusions

In conclusion thyroid hormones exert a key role in the modulation of antioxidant systems and OS is demonstrated both in hyper- and hypothyroidism. In the field of hypothyroidism, a debated question is the treatment of NTIS. Even if in the literature data are conflicting [67,112,113], our data suggest to consider NTIS as a real hypothyroidism at tissue level and not only as an adaptive response to the conditions mentioned above. In particular, CoQ_10_ levels seem to be a reliable index of thyroid hormone effects; moreover, OS is a mechanism to be underlined in the physiopathology of NTIS and, again, it can reflect a condition of hypothyroidism. The question of usefulness of replacement therapy is complex and based on standardization of different factors involved: the choice of hormone (T4 or T3); the route of administration (oral or *i.v.*); and the definition of clinical endpoints, due to the complexity of clinical models with different interfering factors.

When the molecular mechanisms underlying low T3 levels are better understood, it may be possible to choose which patients are likely to benefit from replacement therapy as well as the appropriate schedule of treatment.
